# Regional zircon U-Pb geochronology for the Maniitsoq region, southwest Greenland

**DOI:** 10.1038/s41597-021-00922-x

**Published:** 2021-05-25

**Authors:** Hugo K. H. Olierook, Christopher L. Kirkland, Julie A. Hollis, Nicholas J. Gardiner, Chris Yakymchuk, Kristoffer Szilas, Michael I. H. Hartnady, Milo Barham, Bradley J. McDonald, Noreen J. Evans, Agnete Steenfelt, Pedro Waterton

**Affiliations:** 1 Timescales of Mineral Systems Group, School of Earth and Planetary Sciences, Curtin University, GPO Box U1987, Perth, WA 6845 Australia; 2 John de Laeter Centre, Curtin University, GPO Box U1987, Perth, WA 6845 Australia; 3 Department of Geology, Ministry of Mineral Resources, Government of Greenland, P.O. Box 930, 3900 Nuuk, Greenland; 4 School of Earth and Environmental Sciences, University of St. Andrews, St. Andrews, KT16 9AL UK; 5 Department of Earth and Environmental Sciences, University of Waterloo, Waterloo, Ontario N2L 3G1 Canada; 6 Department of Geosciences and Natural Resource Management, University of Copenhagen, Øster Voldgade 10, 1350 Copenhagen, Denmark; 7 Geological Survey of Denmark and Greenland, Øster Voldgade 10, 1350 Copenhagen K, Denmark

**Keywords:** Precambrian geology, Tectonics, Geochemistry

## Abstract

Zircon U-Pb geochronology places high-temperature geological events into temporal context. Here, we present a comprehensive zircon U-Pb geochronology dataset for the Meso- to Neoarchean Maniitsoq region in southwest Greenland, which includes the Akia Terrane, Tuno Terrane, and the intervening Alanngua Complex. The magmatic and metamorphic processes recorded in these terranes straddle a key change-point in early Earth geodynamics. This dataset comprises zircon U-Pb ages for 121 samples, including 46 that are newly dated. A principal crystallization peak occurs across all three terranes at ca. 3000 Ma, with subordinate crystallization age peaks at 3200 Ma (Akia Terrane and Alanngua Complex only), 2720 Ma and 2540 Ma. Metamorphic age peaks occur at 2990 Ma, 2820–2700 Ma, 2670–2600 Ma and 2540 Ma. Except for one sample, all dated metamorphic zircon growth after the Neoarchean occurred in the Alanngua Complex or within 20 km of its boundaries. This U-Pb dataset provides an important resource for addressing Earth Science topics as diverse as crustal evolution, fluid–rock interaction and mineral deposit genesis.

## Abstract (summary)

**Table:** 

Measurement(s)	sediment deposition • magma • crystallization • metamorphism
Technology Type(s)	laser ablation inductively coupled plasma mass spectrometry • secondary ion mass spectrometry
Sample Characteristic - Environment	rock • outcrop
Sample Characteristic - Location	Maniitsoq • Greenland

Machine-accessible metadata file describing the reported data: 10.6084/m9.figshare.14178398

## Background & Summary

Geochronology aims to establish the timing of geological events using naturally-occurring radioactive isotopes. The U–Pb isotopic system is the benchmark for determining the age of geological materials because, unlike other chronometers, it exploits two independent isotopic decay schemes, allowing open-system behavior (i.e., radiogenic-Pb loss) to be detected and it permits accurate evaluation of temporal context (i.e., time of mineral growth versus evidence of subsequent disturbance). Minerals whose crystal structure rejects Pb are preferable for U-Pb geochronology because they dominantly contain radiogenic Pb produced from *in situ* decay. Zircon is by far the most commonly utilized mineral for U–Pb dating as it contains sufficient U for robust age determination, rejects common (or non-radiogenic) Pb and is an alteration-resistant mineral in crustal rocks.

The North Atlantic Craton, of which the Maniitsoq region in SW Greenland forms a key component, has been fundamental for research into a range of tectonic, magmatic, planetary and economic processes. In the Maniitsoq region, the Akia Terrane is the dominant crustal unit, forming one of the largest components within the North Atlantic Craton^1^ and representing one of the largest well-exposed blocks of Mesoarchean deep crust on the planet^2–4^. The Akia Terrane dominantly comprises rocks with crystallization ages that fall into two distinct age and lithological groupings: (i) a dioritic core formed at ca. 3230–3190 million years ago (Ma), and (ii) voluminous 3070–2970 Ma tonalitic crust^5,6^. Equivalent ca. 3 billion year old (Ga) crust is also recorded in the Alanngua Complex and Tuno Terrane, both north of the Akia Terrane. Several other crystallization and metamorphic events occurred in the Maniitsoq region. Eo- to Paleoarchean detrital zircon grains (4.0–3.2 Ga) found in stream sediments indicate an ancient component present in the unsampled northeastern parts of the region^7^. After ca. 2970 Ma, at least a portion of the supracrustal rocks in the Maniitsoq region were deposited onto Mesoarchean basement between ca. 2880 and 2860 Ma, buried and metamorphosed between 2800 and 2700 Ma, and partially melted at ca. 2730 Ma^8,9^. Further greenschist to amphibolite-facies metamorphic events occurred at ca. 2630 Ma, as recorded by metamorphic zircon overgrowths and neoblastic apatite^10^, and 2540 Ma, as evidenced from zircon^11^ and titanite^12^. Both of these late-stage events may be related to protracted terrane assembly^13,14^.

Geochronology in the Maniitsoq region has aimed to address several fundamental geological questions. Perhaps the most important of these is the nature of early crustal growth, and whether this is linked to some form of subduction, which might in turn reflect early plate tectonics^4,15,16^. On the early Earth, the primary mode of crustal growth and recycling may have been within a volcanic plateau-type setting, perhaps driven by mantle upwellings^17^, density foundering and melt generation^18^. Exactly when the switch from dominantly vertical to horizontal tectonics occurred continues to be debated but there is increasing support for a global transition around 3.2 to 3.0 Ga^7,19–25^. The Akia Terrane has also been proposed to host an unconfirmed ca. 3.0 Ga impact structure^26–29^ but this proposal is not widely accepted^30^ and has been questioned on a range of grounds^8,9,31–35^. The processes of Meso- to Neoarchean magmatic Ni–Cu ± Cr–platinum group element systems^33,36^ and Neoarchean orogenic Au^37,38^ are also nascent in SW Greenland^33,39^.

**Fig. 1:**
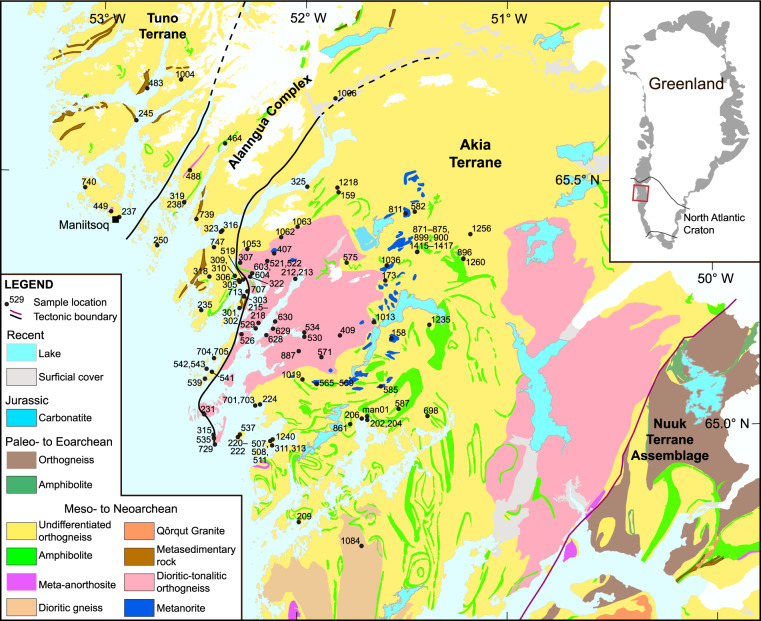
Simplified geological map of the Maniitsoq region with sample locations labelled. Map is modified from Gardiner, *et al*.^15^, Olierook, *et al*.^64^ and Steenfelt, *et al*.^9^. Map projection is WGS 1984, UTM Zone 22 N. Note, samples ev01 to ev04 plot to the north of the map and are omitted for clarity.

Due to the numerous questions that can be addressed with zircon geochronology of the Maniitsoq region, there have been a number of papers published on the ages of components in the Akia Terrane and its bordering tectonic belts (including the Tuno Terrane and Alanngua Complex) in the past four decades (see Data Records). Despite its importance, a central repository for modern zircon U–Pb geochronology data is lacking for the Maniitsoq region. Here, we present a record of 121 samples with recent zircon U-Pb geochronological data, either published in the Maniitsoq region (75 samples) or newly reported (46 samples; see sample locations in Fig. 1). All data were collected as part of the wider Maniitsoq Mapping project, funded by the Ministry of Mineral Resources, Greenland, and were produced through a workflow with standardized isotopic outputs. If available, sample aliquots or residues may be requested from the Ministry of Mineral Resources, Greenland. Ultimately, zircon geochronology in the Maniitsoq region provides a regionally and potentially globally important resource to help understand the geological development of this section of crust and processes that operated on the Archean Earth.

## Methods

### Analytical methods for new samples

Approximately 5 kg of each sample was crushed and the resultant slurries were put through a Wilfley concentrating shaker table for initial concentration of heavy minerals, which were subsequently separated using LST heavy liquids at 2.9 g cm^−3^. The non-magnetic heavy fraction was isolated using a Frantz isodynamic magnetic separator. Heavy mineral grains were subsequently hand-picked from the non-magnetic fraction, mounted in 25 mm epoxy rounds together with zircon reference materials and polished to approximately half grain thickness to expose grain interiors.

Each mount was imaged using transmitted and reflected light to provide internal grain textural information. Cathodoluminescence (CL) imaging was conducted using a Mira3 Field Emission Gun SEM (FEG-SEM) at the John de Laeter Centre (JdLC), Curtin University. Cathodoluminescence images were used to document internal zonation patterns (e.g. oscillatory, sector, patchy), identify recrystallization textures and recognize the presence of any crystal rims. These imaging procedures aid in elucidating the zircon growth processes (e.g. magmatic [igneous], metamorphic, or disturbed – recrystallized, e.g.^40^).

For LA-ICP-MS data, zircon U-Pb data were collected at the GeoHistory Facility, JdLC, Curtin University, across 15 sessions. Where possible, multiple spots were collected from both grain cores and rims. An excimer laser (RESOlution LR or LE 193 nm ArF with a Lauren Technic S155 cell) with spot diameters of 15–50 µm, on-sample energy of ~2.3 J cm^−2^, and a repetition rate of 5 Hz was used to sputter target zircon for 15–45 seconds of analysis time and 25–60 seconds of background capture. All analyses were preceded by two cleaning pulses. The sample cell was flushed by ultrahigh purity He (0.34–0.68 L min^−1^) and N_2_ (1.2–2.8 mL min^−1^). U-Pb data were collected on either an Agilent 7700 s single quadrupole, 8900 triple quadrupole or Nu Plasma II multi-collector mass spectrometers with high purity Ar as the carrier gas (flow rate = 0.98 L min^−1^). Analyses of every ~20 unknowns were bracketed by analyzing a standard block containing the primary zircon reference material OG1 (3465.4 ± 0.6 Ma)^41^, which was used to monitor and correct for mass fractionation and instrumental drift. OG1 was chosen as the primary reference material due to its similar age and ablation response to the unknown analyses of the Maniitsoq region, which have ages dominantly between 3.8 and 2.5 Ga. The standard block also contained Plešovice (337.13 ± 0.37 Ma)^42^, GJ-1 (601.95 ± 0.40)^43,44^, 91500 (1063.78 ± 0.65 Ma)^44,45^ and Maniitsoq (3008.70 ± 0.72 Ma)^46^ (all uncertainties at 2 standard deviations from the mean; 2σ), which were used as secondary reference materials to monitor data accuracy and precision. Validation of these reference materials during each analytical run is presented in the Technical Validation section. All LA-ICP-MS Data were reduced using Iolite3 or 4^47^ and in-house Excel macros.

For SIMS analyses, U-Pb data were collected using the Sensitive High Resolution Ion Micro Probe Facility, JdLC, Curtin University, across 12 sessions. The spot size across all sessions was ~22 × 16 µm. Prior to analysis, each site was cleaned by rastering the primary ion beam over the target area for up to 2.5 minutes. U-Pb ratios and absolute abundances were determined relative to the CUYZ standard zircon (^206^Pb/^238^U age = 568.55 Ma; ^207^Pb/^206^Pb age = 569.49 Ma; U = 582.7 ppm; Th = 82.7 ppm^48,49^) or 91500 (1063.78 ± 0.65 Ma^44,45^). Primary reference material analyses were interspersed with those of unknown zircons and secondary reference materials Plešovice (337.13 ± 0.37 Ma^42^) or OG1 (3465.4 ± 0.4 Ma^41^). Validation of these reference materials during the analytical run is presented in the Technical Validation section. Fractionation of ^206^Pb*/^207^Pb* (Pb* = radiogenic Pb) was monitored and no fractionation correction was deemed necessary for any session. Measured compositions were corrected for common Pb using measured ^204^Pb. In most cases, corrections are sufficiently small to be insensitive to the choice of common Pb composition, and an average crustal composition^50^ appropriate to the age of the mineral was assumed, as generally common Pb counts did not fall during the analyses (i.e., common Pb was not surface derived). Data were reduced using SQUID^51^, in-house macros, and Isoplot^52^, using decay constants of Steiger and Jäger^53^ and ^238^U/^235^U ratios of Hiess, *et al*.^54^.

### Filtering of new and previously published geochronological data

A compilation of new and recently published zircon U-Pb data is collated and placed into a standardized format for U-Pb geochronology as recommended by Horstwood, *et al*.^44^ (see Data Records). All collated analytical data were collected under a unified workflow and is thus amenable to standardisation, a prerequisite to judging the accuracy and precision (see Technical Validation). We note that due to slight differences in filtering between this study and previously published studies (particularly Step 3), there may be slight differences in published ages and ages presented in this compilation. However, for the vast majority of analyses, this age difference is less than the 2σ uncertainty on the age data, and the age difference is therefore statistically insignificant. All zircon U-Pb data presented herein were collected using *in situ* ablation techniques, either secondary ion mass spectrometry (SIMS) or laser ablation inductively coupled plasma mass spectrometry (LA-ICP-MS; see data record^55^ for sample information).

#### Step 1 – Removal of mixed analyses

All analytical data was first categorized on the basis of linking the analytical location to interior texture images to allow the identification of inadvertent sample mixtures (e.g. core-rim mixtures^56^).

#### Step 2 – Correction of common Pb

The identification and correction of any incorporated ‘common’ ^206^Pb, ^207^Pb and ^208^Pb during crystallization of zircon (i.e., not from the radiogenic decay of ^238^U, ^235^U and ^232^Th) is treated differently for SIMS and LA-ICP-MS data as required by the analytical approach.

For zircon SIMS data, the incorporation of any Pb during crystallization (i.e., common-Pb or Pb_c_) was corrected for by monitoring ^204^Pb and correcting ^206^Pb and ^207^Pb using this value in reference to the terrestrial Pb model after Stacey and Kramers^50^. This ^204^Pb correction used assumed contemporaneous Pb according to this model. Zircon analyses with >1% common-Pb (*f*206%) are considered to have a large common Pb correction that may impact on age precision and are thus identified as Group D in the associated data tables.

For zircon LA-ICP-MS data, the interference of ^204^Hg in the carrier gas on ^204^Pb means that low amounts of common-Pb are difficult to measure with sufficient accuracy for a useful correction to be applied using the ^204^Pb approach. Consequently, common-Pb was not corrected for LA-ICP-MS analyses, but rather ^204^Pb was used as a qualitative indicator of the presence of common Pb. To avoid analyses with high common-Pb in LA-ICP-MS data, we considered analyses where the *f*206%, minus the uncertainty on *f*206%, exceed 1% to be excessively influenced by the presence of common Pb and thus identified as Group D.

#### Step 3 – Filtering for concordant analyses

All individual analyses are filtered for concordance. Analyses are considered concordant where the 2σ confidence error ellipse for an analysis intersects the concordia curve in conventional concordia space (i.e., Wetherill), excluding uncertainties on the decay constant^57–60^. All discordant analyses are also included in Group D (discordant).

#### Step 4 – Within-run heterogeneity

Analyses within the 2σ concordance limit but with elevated analytical uncertainties may be a function of a heterogeneous ablation signal during sample analysis, and are also assigned to Group D. We consider >10% uncertainty at 22σ on either the ^206^Pb/^238^U or ^207^Pb/^206^Pb ratios as an indication of signal heterogeneity.

#### Step 5 – Calculation of dates and uncertainties

For concordant data with low *f*206%, all calculated ages are presented based on ^207^Pb/^206^Pb ratios, as these have superior precision to ^206^Pb/^238^U ratios at the typical age range of ca. 3.8–2.5 Ga encountered in the Maniitsoq region^58^. Four of the >3200 analyses are younger than 700 Ma (sampled from kimberlites) and, for these, their ages are determined via ^206^Pb/^238^U ratios given the superior precision on this ratio at these ages. Dates on individual analyses are computed using the decay constants of Jaffey, *et al*.^61^, recommended in Steiger and Jäger^53^. As ^235^U is not measured due to its low abundance, a ^238^U/^235^U ratio of 137.818 is assumed after Hiess, *et al*.^54^. All uncertainties on individual analyses are presented as 2σ, with ages calculated at 95% confidence. All uncertainties on individual analyses include internal and external components of error propagated in quadrature.

#### Step 6 – Assignment of genetic interpretations and computation of geologically-meaningful ages

Concordant data are separated into different genetic groups based upon textural evidence (e.g., zoning in CL^40^) or chemical data (e.g., Th/U ratio, U concentrations^62,63^).

In rocks with an igneous protolith, these genetic groups are coded as follows. Group I; igneous/magmatic, defined as those formed during magmatic crystallization in an igneous rock. Group X; inherited (xenocryst), incorporated from a deeper reservoir or assimilated from the wall-rock during magma ascent. Inherited grains were identified from CL response (e.g., core–rim relationships) or dates that were significantly older than the main cluster of Group I grains^64^. Group M; secondary age reflecting metamorphic processes in the rock, formed either as neoblastic grains or as overgrowths on older cores. Group P; concordant analysis but interpreted to have undergone (partial) loss of radiogenic Pb, as evident from correlations between U and age, alpha dose and age or textural evidence (e.g. fading / blurring of primary zoning). The chance of a zircon grain having undergone (partial) loss of radiogenic Pb through recoil-induced alpha radiation damage (and eventual metamictization) may be mathematically constrained after the equations of Murakami, *et al*.^65^, a function of actinide (U + Th) concentrations and age. Although useful, intermediate to high metamictization states do not necessitate (partial) loss of radiogenic Pb, but it provides a qualitative indicator, together with textural data, to distinguish Group P from other genetic groups.

In rocks with sedimentary protoliths, Groups are defined as follows. Group S; detrital age, derived from the weathering, erosion and transport of older igneous or metamorphic rocks. Group Y; youngest detrital grains. Group Y has a genesis identical to Group S but is defined specifically as the youngest cluster of analyses in a (meta)sedimentary rock (excluding Groups M and P, where applicable), given the importance of this age in defining a maximum depositional age constraint. Whilst there are several different ways in which Group Y can been calculated (e.g. the youngest group or youngest individual analysis)^58^, here, we define Group Y as the youngest cluster of analyses that approaches a mean square of weighted deviates (MSWD) of 1 to account for analytical scatter of a normally-distributed dataset^66^. Like in samples with igneous protoliths, Groups M (metamorphic) and P ([partial] loss of radiogenic Pb) may also be identified in (meta)sedimentary rocks (see above).

For Groups X, M, and I, suffix numbers 1, 2…n are used to describe multiple discrete inherited reservoirs, magmatic events (from intermingling of intrusions) and metamorphic events, respectively. The use of a question mark suffix to any group indicates that any textural or chemical based grouping is uncertain.

Crystallization, metamorphism, maximum emplacement, and maximum depositional ages are calculated from the ^207^Pb/^206^Pb dates of igneous/magmatic (Group I), metamorphic (Group M), inherited (Group X) and youngest detrital analyses (Group Y), respectively (data record^55^). Uncertainties on ages are presented at 2σ, including the analytical (internal) uncertainties, and systematic uncertainties on the primary reference material (0.017% on the standard OG1), the long-term excess variance of validation materials (2% on ^206^Pb/^238^U, 0.5% on ^207^Pb/^206^Pb) and, for SIMS data only, the uncertainty in common Pb correction^50^. All these uncertainties are propagated in quadrature. Decay constant uncertainty is not included in the error propogation as all analyses compare the same isotopic system (U-Pb). If additional isotopic systems are compared to this data set an additional 0.14% (uncertainty on ^207^Pb/^235^U = 0.136% and ^238^U/^235^U = 0.033%, propagated in quadrature) is required to compare ^207^Pb/^206^Pb ages to other ages.

#### Step 7 – Visualizing of data

**Fig. 2:**
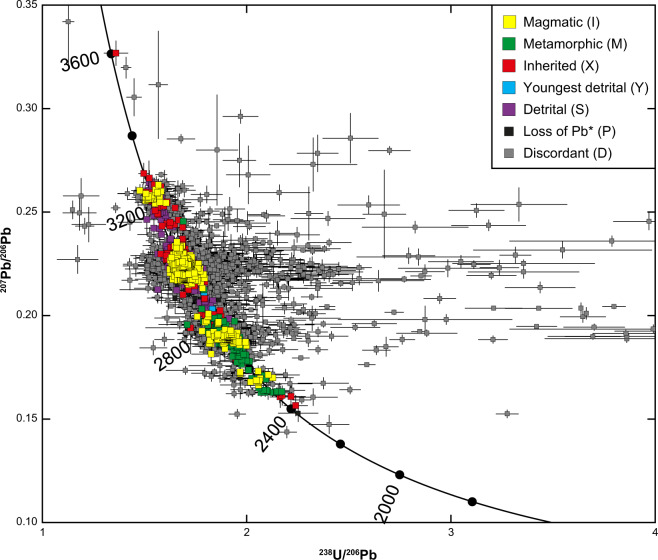
Tera-Wasserburg inverse concordia plot of all data in this study. Highly discordant data (i.e., ^238^U/^206^Pb > 4 and/or ^207^Pb/^206^Pb > 0.035) and kimberlite data (Phanerozoic) are not shown for clarity.

**Fig. 3:**
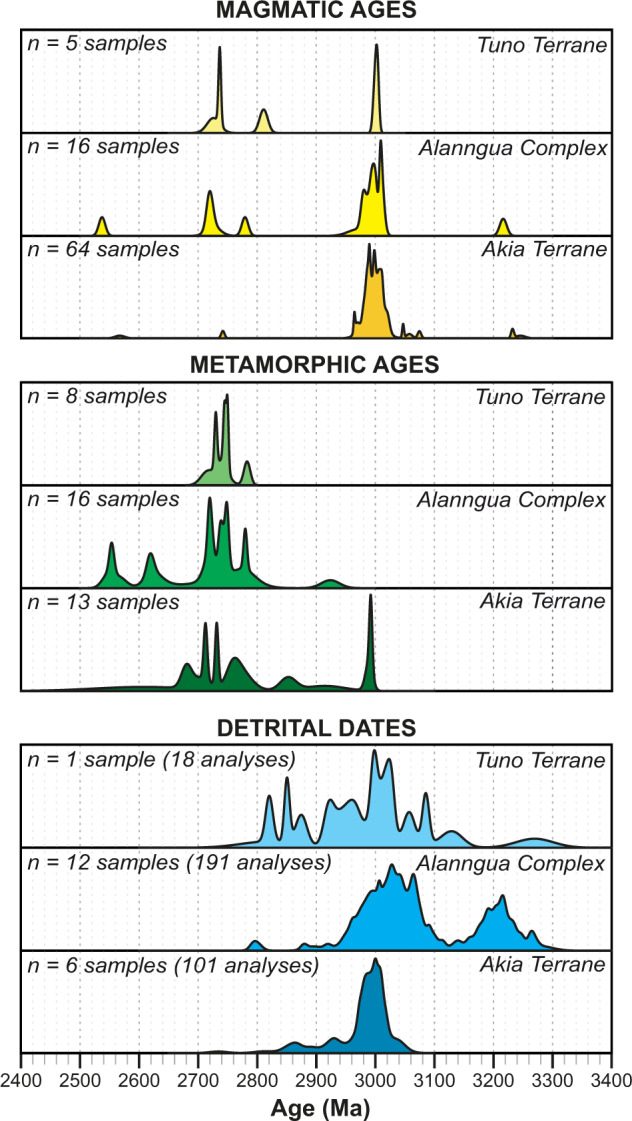
Probability density plots of magmatic ages, metamorphic ages and detrital dates for the Tuno Terrane, Alanngua Complex and Akia Terrane.

**Fig. 4:**
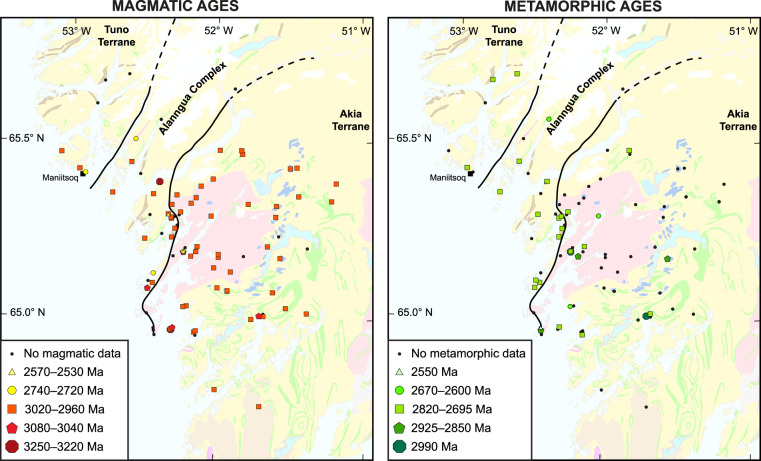
Maps of the magmatic and metamorphic ages in the Maniitsoq region. A semi-transparent geological map from Fig. 1 is laid under the color-coded age symbols. (**a**) Crystallization ages of magmatic samples (Group I). (**b**) Metamorphic ages of samples with metamorphic events (Group M). For clarity, kimberlites (Phanerozoic) are not shown.

Three sets of data visualization are presented. The first of these is a Tera-Wasserburg^67^ inverse concordia plot that shows the distributions of concordant and discordant age data, and interpretations attached to concordant data (Fig. 2). The second visualization technique involves plotting magmatic crystallization ages, metamorphic event ages and detrital dates as probability density plots for the Akia Terrane, Alanngua Complex and Tuno Terrane, using Isoplot v4.15^52^ (Fig. 3). The final visualizations are magmatic crystallization ages and metamorphic events, colour-coded and symbol-coded by their distinct events (Fig. 4). Boundaries between different groupings occur at natural breaks in the data, as identified from Fig. 3.

## Data Records

The data record in this contribution includes four different datasets, and a series of maps and graphs that illustrates the distribution of data, which may be found in the data repository associated with this article^55^.

The first of the four datasets is a summary U-Pb data table of all new and published samples with sample names, coordinates (all in WGS 1984 Geodetic), analyzed mineral, instrument, date of analysis and columns related to the age of the sample. For rocks with igneous protoliths, these age-based columns provide information as to the crystallization age, inherited components and any metamorphic event ages. For rocks with sedimentary protoliths, the age-based columns provide maximum depositional ages, principal detritus age ranges and any metamorphic event ages.

The second of the datasets provides a complete data table of all new and recently published U-Pb analyses from the Maniitsoq region in the format recommended by Horstwood, *et al*.^44^ with additional information on genetic interpretation and CL zone and response.

The third of the datasets is a compilation of CL images, annotated by spot number, which provide textural information of analyses.

For visualization of data, we provide maps with annotations of crystallization and metamorphism ages (Fig. 4). For crystallization ages in igneous rocks, metamorphic events and detrital grains in sedimentary rocks, we provide probability density plots (Fig. 3).

## Technical Validation

A key aspect of *in situ* U-Pb geochronology is validation of unknown analyses by evaluating the statistical validity of co-analyzed reference materials with known ages for each analytical run. For both SIMS and LA-ICP-MS, a primary (or external) reference material is used to correct for downhole fractionation, fractionation of elements and their oxides, and instrumental drift^44^.

For the vast majority of LA-ICP-MS analyses in this study (including all new analyses), the primary reference material zircon OG1, with a published ^207^Pb/^206^Pb age of 3465.4 ± 0.4 Ma^41^, is the most appropriate matrix-matched reference material to the ca. 3.8–2.5 Ga unknowns of the Maniitsoq region. In one analytical session, zircon reference material GJ-1 was used as a primary reference material due to insufficient analyses of OG1 in this run (29/09/2017). To validate the analytical procedure and monitor data accuracy and precision, one or more secondary (or internal) reference materials was used^44^. For sessions in 2017–2018, zircon reference materials Plešovice (337.13 ± 0.37 Ma^42^), GJ-1 (^206^Pb/^238^U age = 601.95 ± 0.40 Ma, ^207^Pb/^206^Pb age = 608.5 ± 1.8 Ma^43,44^) and/or 91500 (1063.78 ± 0.65 Ma^44,45^) were used (all uncertainties at 2σ). For more recent analytical sessions in 2019, a newly developed reference material, Maniitsoq (3008.70 ± 0.72 Ma^46^), derived from megacrysts in the Akia Terrane, was primarily used as a secondary reference material due to the similar ablation response to OG1 and the ~3 Ga unknown grains. However, the aforementioned well-established reference materials Plešovice, GJ-1 and 91500 were also co-analyzed to validate the ^206^Pb/^238^U ratios. All secondary reference materials, listed next to the primary reference material, for each analytical run are provided in the data record^55^. In all cases, the weighted mean ^206^Pb/^238^U or ^207^Pb/^206^Pb ages of secondary reference materials overlap within uncertainty at 2σ, with a probability of fit ≥0.05 for the chi-squared test, satisfying the null hypothesis that they constitute a single group, including Plešovice (335.6 ± 1.6 to 340 ± 10 Ma [^206^Pb/^238^U ages]), GJ-1 (596 ± 10 to 606.5 ± 4.4 Ma [^206^Pb/^238^U ages] and 603 ± 14 Ma to 615 ± 6 Ma [^207^Pb/^206^Pb ages]), 91500 (1054 ± 10 to 1077 ± 14 Ma [^206^Pb/^238^U ages]) and Maniitsoq (3000 ± 8 to 3015 ± 8 Ma [^207^Pb/^206^Pb ages]).

For SIMS analyses, U-Pb ratios and absolute abundances were determined relative to the CUYZ reference zircon (^206^Pb/^238^U age = 568.55 Ma; ^207^Pb/^206^Pb age = 569.49 Ma; U = 582.7 ppm; Th = 82.7 ppm^48,49^) or 91500 standard zircon (1063.78 ± 0.65 Ma; U = 71–86 ppm; Th/U = 0.34^44,45^). Primary reference materials were interspersed with those of unknown zircons and secondary reference materials Plešovice (337.13 ± 0.37 Ma^42^) or OG1 (3465.4 ± 0.4 Ma^41^). All primary and secondary standards yielded statistically reliable (*p* > 0.05) weighted mean analyses, including CUYZ (566.0 ± 2.7 to 571 ± 8 Ma), 91500 (1059 ± 9 and 1063 ± 22 Ma), Plešovice (334.0 ± 3.9 to 341 ± 8 Ma) and OG1 (3460 ± 9 to 3471 ± 8 Ma), all of which overlap with published ages within 2σ uncertainty.
